# Systematic Characterization and Comparative Analysis of the Rabbit Immunoglobulin Repertoire

**DOI:** 10.1371/journal.pone.0101322

**Published:** 2014-06-30

**Authors:** Jason J. Lavinder, Kam Hon Hoi, Sai T. Reddy, Yariv Wine, George Georgiou

**Affiliations:** 1 Department of Chemical Engineering, University of Texas at Austin, Austin, Texas, United States of America; 2 Institute for Cellular and Molecular Biology, University of Texas at Austin, Austin, Texas, United States of America; 3 Department of Biomedical Engineering, University of Texas at Austin, Austin, Texas, United States of America; 4 Section of Molecular Genetics and Microbiology, University of Texas at Austin, Austin, Texas, United States of America; Institut de Recherches Cliniques de Montréal (IRCM), Canada

## Abstract

Rabbits have been used extensively as a model system for the elucidation of the mechanism of immunoglobulin diversification and for the production of antibodies. We employed Next Generation Sequencing to analyze Ig germline V and J gene usage, CDR3 length and amino acid composition, and gene conversion frequencies within the functional (transcribed) IgG repertoire of the New Zealand white rabbit (*Oryctolagus cuniculus*). Several previously unannotated rabbit heavy chain variable (VH) and light chain variable (VL) germline elements were deduced bioinformatically using multidimensional scaling and k-means clustering methods. We estimated the gene conversion frequency in the rabbit at 23% of IgG sequences with a mean gene conversion tract length of 59±36 bp. Sequencing and gene conversion analysis of the chicken, human, and mouse repertoires revealed that gene conversion occurs much more extensively in the chicken (frequency 70%, tract length 79±57 bp), was observed to a small, yet statistically significant extent in humans, but was virtually absent in mice.

## Introduction

B cell development and repertoire diversification vary significantly among vertebrate species [1]. Diversification of the Ig repertoire occurs through the combinatorial joining of numerous V, D, and J gene segments for the Ig heavy chain (or just V and J gene segments in the case of Ig light chains) through several mechanisms collectively referred to as VDJ recombination, followed by somatic mutagenesis upon subsequent B-cell encounter with foreign antigen. Compared to humans and mice, which use a diverse assortment of germline VH gene segments during VDJ recombination of the heavy chain, the rabbit IgH repertoire displays highly restricted VH gene segment usage. Earlier studies had indicated that the majority of B cells in the rabbit utilize the VH1 gene, the most D-proximal VH locus [2]. VH1 Ig are serotypically VHa-positive, and there are three distinct VHa allotypic lineages (a1, a2, and a3) [3], [4]. In addition, approximately 10–20% of expressed Ig in rabbits are serotypically VHa-negative (VHn) [4], [5]. The VHn Ig genes that have been annotated in rabbits (VHx, VHy, and VHz) are encoded by loci significantly upstream (>100 kb) of the VH1 gene locus [6]. Recently, sequencing of the rabbit genome has enabled the identification of germline Ig elements in a Thorbecke inbred rabbit [7]. Overall, >300 VH-like gene sequences were identified within 79 unplaced genomic scaffolds (i.e. unknown chromosomal locations). The large number of previously unannotated VH-like sequences identified within the a1/a2 Thorbecke rabbit, as well as previously identified sequences from latent heavy chain allotypes [2], [8], clearly demonstrate the complexity of the germline Ig repertoire. However, because the sequenced Thorbecke rabbit was heterozygous at the IgH locus (a1/a2 based on mapping of the VH1 gene), the actual number of distinct VH gene elements in the haploid genome is unclear.

Another major source of Ig repertoire diversity derives from the somatic introduction of non-templated nucleotides into the imprecise junctions formed by the variable ligation of recombining V-D and D-J gene segments—a process known as N-nucleotide addition. This hypervariable V-*N*-D-*N*-J interval defines CDR3 of the heavy chain (CDRH3). Species such as cattle have extremely long CDRH3s [9] as a result of increased levels of N-nucleotide addition. Longer CDRH3s not only create a more expansive and diverse sequence space in the Ig repertoire, but may also hold unique functional relevance in protection against disease [10]. For most mammalian species, N-nucleotide addition during VJ recombination of the light chain is limited and therefore junctional diversity in the light chain is much less pronounced compared to the heavy chain; however, rabbits have been shown to have light chain CDR3s (CDRL3s) that are unusually longer and more diverse, indicating significant N-nucleotide addition during light chain VJ recombination [11].

After VDJ recombination, the naïve Ig repertoire in rabbits is further diversified in the first 2 months of age by extensive somatic mutagenesis in the gut-associated lymphoid tissue (GALT) [12], through both somatic hypermutation (SHM) and gene conversion events [13], both of which have been shown to be dependent upon the exposure of the naïve B cell repertoire to the gut microflora [14]. Ig gene conversion is employed not only by rabbits, but also by other species including chickens and involves the non-reciprocal homologous recombination of upstream donor V gene loci into the recombined VDJ (and VJ) locus. Like SHM, Ig gene conversion is mediated through the enzyme *a*ctivation *i*nduced cytidine *d*eaminase (AID) [15] and thus is often found to occur proximal to hotspot AID motifs conserved within germline V genes. In chickens, gene conversion has been shown to be the dominant mechanism of AID-mediated mutagenesis [16] and involves a single functional VH and VL gene undergoing gene conversion with numerous upstream VH and VL pseudogenes, respectively [17]. In rabbits, however, the upstream loci are a mix of functional V genes and pseudogenes that can serve as potential donor sequences in gene conversion events. The fundamental properties of gene conversion events and the relative extent to which gene conversion plays a role in rabbit Ig diversification is not entirely clear, mostly due to limitations in sampling and difficulty in precise, automated identification of gene conversion events in highly mutated Ig sequences.

Here, we present a thorough characterization of the expressed rabbit IgG repertoire. We identify several unannotated functional rabbit germline VH and VL germline gene sequences and provide a comprehensive survey of the salient features of the rabbit Ig repertoire. We estimate the gene conversion frequency in the rabbit and demonstrate that it is significantly less than that observed in the chicken repertoire and, not surprisingly, much greater than that observed in humans and mice.

## Materials and Methods

### Ethics Statement

Three New Zealand white (NZW) rabbits and one white leghorn chicken were used for this work, as approved through the Institutional Animal Care and Use Committee (IACUC) of the University of Texas at Austin (protocol AUP-2011-00016). All efforts were made to ensure animal welfare and minimize suffering in accordance with the United States Department of Agriculture (USDA) Animal and Plant Health Inspection Service (APHIS) Guidelines for animal care and husbandry.

### Isolation of B cells from immunized rabbits, chicken, mouse, and human

At sacrifice, rabbit femoral bone marrow (BM) cells were isolated and approximately 100 ml blood was collected into heparin tubes. Blood aliquots of 20 ml were gently layered over 20 ml of Histopaque 1077 (Sigma, MO, USA) and centrifuged in a swinging bucket rotor at 400 *g*, 45 min at 25°C (Beckman Coulter). The serum was removed from the top of the gradient and stored at −20°C. PBMCs were isolated from the intermediate layer. Each collected tissue (BM and PBMC) was processed as previously described [18], with the exception that the PBMCs did not require red blood cell lysis after gradient centrifugation. CD138^+^ cells were isolated as previously described [19]. PBMCs or CD138^+^ BM plasma cells (PCs) were centrifuged at 930×*g*, 5 min at 4°C. Cells were then lysed with TRI reagent (Ambion, TX, USA) and total RNA was isolated according to the manufacturer's protocol in the Ribopure RNA isolation kit (Ambion). RNA concentrations were measured with an ND-1000 spectrophotometer (Nanodrop, DE, USA).

For the chicken, total RNA was prepared from splenic tissue of a white leghorn chicken using TRIzol reagent (Life technologies) and purified with RNeasy Micro Kit (Qiagen, CA). cDNA was generated from total RNA using oligo(dt) according to the manufacturer's protocol (Superscript II First strand Synthesis kit, Life Technologies), PCR-amplified as described previously [20] using chicken IgY-specific primers listed in Table S1, and sequenced using the 2×250 paired end MiSeq Next Generation Sequencing (NGS) platform (Illumina, San Diego, CA). The two IIlumina 2×250 output files were aligned using FLASH [21] and CDRH3 and full-length VH sequences were determined using in-house probabilistic model [18] for delimiting the CDRH3 regions based on *Gallus gallus* Ig sequences found in NCBI Genbank.

### Amplification and high-throughput sequencing of rabbit VH and VL gene repertoires

Approximately 0.5 µg of ethanol precipitated RNA was used for first-strand cDNA synthesis according to the manufacturer's protocol for 5′ RACE using the SMARTer RACE cDNA Amplification kit (Clontech, CA, USA). The cDNA reaction was diluted into 100 µl of Tris-EDTA buffer and stored at −20°C. 5′ RACE PCR amplification was performed on the first strand cDNA to amplify the VH repertoire with the kit-provided, 5′ primer mix and 3′ rabbit IgG-specific primers RIGHC1 and RIGHC2 (Table S1). The rabbit VL repertoire was amplified via 5′ RACE, using a 3′ primer mix specific for both the Vκ and Vλ rabbit constant regions. The VL primers comprised 90% RIGκC mix and 10% RIGλC mix (Table S1) to approximate known ratios of light chain isotypes in rabbits. Reactions were carried out in a 50 µl volume by mixing 35.25 µl H_2_O, 5 µl 10X Advantage-2 PCR buffer (Clontech), 5 µl 10X Universal Primer A mix (Clontech), 0.75 µl Advantage-2 polymerase mix (Clontech), 2 µl cDNA, 200 nM V_H_ or V_L_ primer mix, and 200 µM dNTP mix. PCR conditions were: 95°C for 5 min, followed by 30 cycles of amplification (95°C for 30 sec, 60°C for 30 sec, 72°C for 2 min), and a final 72°C extension for 7 min. The PCR products were gel-purified to isolate the amplified VH or VL DNA (∼500 bp). 100 ng of each 5′ RACE amplified VH or VL DNA was processed for Roche GS-FLX 454 DNA sequencing according to the manufacturer's protocol. The 454 dataset has been deposited at the NIH SRA (Sequence Read Archive) under accession number SRP042296.

All 454 data were first processed using the sequence quality and signal filters of the 454 Roche pipeline and then subjected to bioinformatics analysis that relied on homologies to conserved framework regions using IMGT/HighV-Quest Tool [22]. Additional filters were applied for full repertoire database construction as follows: (i) Length cutoff: full-length sequences were filtered by aligned amino acid lengths >70 residues and aligned framework 4 region lengths >2 residues; (ii) Stop codons: aligned amino acid sequences containing stop codons were removed.

### IgBLAST alignment, Multidimensional scaling (MDS), and k-means analysis

An IgBLAST database for germline annotation of the rabbit IgG sequences was constructed using the following sequences: the IMGT rabbit V germline reference set that includes the allotypic a2 sequences in BAC clones AY386694 and AY386697 [23], allotypic a2 sequences from an Alicia rabbit (AF176997 through AF177016) [24], potentially latent IGHV (M12180, M60121, M60336) [8], [25], [26], allotypic a1 sequences VH1-a1 (M93171), VH3-a1 (M93177), and VH4-a1 (M93181) [27], and the allotypic a3 sequences VH1-a3 through VH7-a3 (M93173, M93176, M93179, M93183, M93184, M93185, M93186) [13], [27]. In addition to the IMGT rabbit reference set, initial IgBLAST database included VH8-a3 through VH11-a3 (L27311, L27312, L27313, L27314) [28], VHx (L03846) [29], and VHy (L03890) [29]. For light chain, the IMGT database was used without addition. IgBLAST alignments against the database were analyzed by bit score (and equivalently the number of called nucleotide mutations per sequence). Aligned (annotated to a certain germline) sequences with greater than 30 called mutations were extracted from this initial IgBLAST alignment and these poorly aligned sequences were aligned using MUSCLE [30] multiple sequence alignment (BLOSUM80 substitution matrix, gap open penalty -15, gap extend penalty -3). For calculating distance matrices and performing MDS, the package bios2mds [31] in the R environment was used. The MUSCLE alignment was imported into R and the pairwise distance matrix calculation using the ‘mat.dif’ function, which computes a distance matrix based on pairwise differences between each sequence was performed. Metric MDS analysis of the pairwise distance matrix was performed using the function ‘mmds’, which reduces the dimensionality of the distance matrix into Euclidean space. These Euclidean values are analyzed by k-means silhouette scoring (function ‘sil.score) and k-mean clustering (function ‘Kmeans’) to identify distinct sets of sequences that each derive from an unannotated germline Ig sequence. The sequences from each cluster are extracted and aligned in MUSCLE. For each derived cluster alignment, the consensus sequence was searched by BLASTn against the non-redundant nucleotide collection and the rabbit genome.

### IMGT and IgBLAST repertoire analyses

Germline V gene assignments were derived from IgBLAST alignments against the database described above. Germline J gene assignments and CDR3 sequences (rabbit, mouse, and human) were derived from IMGT HighV-Quest alignments. Chicken CDR3 sequences were derived from a position weight matrix motif search of the FR3 and J region in chickens.

### Gene conversion analysis

For rabbits, IgBLAST alignments of the NGS data sets was performed using custom BLAST databases for rabbit, as detailed above. For the chicken, the IgBLAST database included the functional VH1 sequence, along with 18 known VH pseudogenes [17]. For mouse and human, the IgBLAST-provided database was used. IgBLAST was used to assign the best-scoring germline VH reference sequence for each query sequence. To detect gene conversion events in the query, the assigned germline reference sequence was then scored against all other germline reference sequences in the IgBLAST alignment as follows: 1) For each VH germline in the alignment (each a possible donor VH sequence) except the assigned one, we used a scoring function that assigns a ‘1’ at each position only if the putative donor VH matches and the assigned reference VH germline mismatches, a ‘0’ at each position that both references either match or both mismatch, and a ‘−1’ at each position that the assigned reference VH matches and the putative donor VH mismatches. 2) Search each scored putative donor VH for stretches of positions that score as ‘1’, with a putative gene conversion event called only if three positions scoring ‘1’ are uninterrupted by positions scoring ‘−1’. The gene conversion event boundaries were defined by positions scoring ‘−1’ (long tract boundary) or by the most distal positions of the tract that score ‘1’ (short tract boundary). Adjacent long tracts from the same donor VH are automatically combined by allowing long tracts with a shared boundary to connect. Positions of the alignment that have gaps in the query are scored as ‘0’ in all putative donor VH scored positions. To exclude PCR crossover products or gene replacement events (single crossover events), all gene conversion events that start within the first 15 positions or end with the last 15 positions of the aligned VH gene are excluded (e.g. the gene conversion must be an internal double crossover event with sufficient sequence from the assigned VH on each side). The donor VH selected represents the germline VH with the highest scoring tract (sum of the tract positional scores). P-values for the gene conversion events are scored as described [32], with the exception that all polymorphic sites are permuted during the permutation test. The p-values described here are local p-values calculated via 1000 iterations of positional permutation of the assigned and donor VH germlines. Only gene conversion events with a p-value below 0.05 (95% confidence interval) and a minimum tract score >4 (to avoid effects of high SHM) are considered as high confidence events.

## Results

### Identification of putative rabbit VH germline elements using multidimensional scaling of high throughput sequencing data

**Figure 1:**
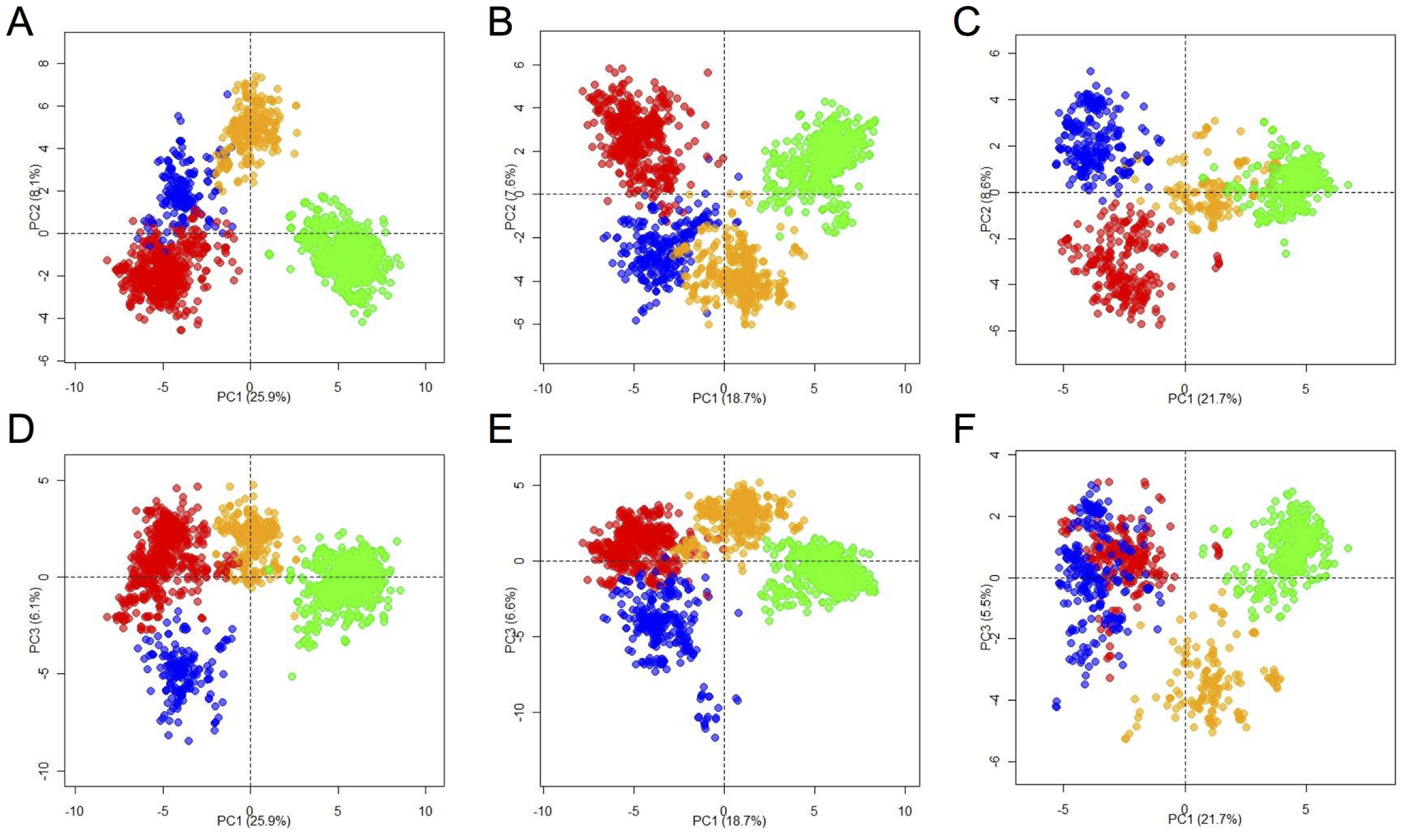
MDS and k-means clustering of low scoring alignments for VH sequences. The first three principle components of the MDS are shown here, with k-means defined clusters colored differently. PC1 (principle component 1) v. PC2 (principle component 2) for (A) rab1, (B) rab2, and (C) rab3, and PC1 v. PC3 (principle component 3) for (D) rab1, (E) rab2, and (F) rab3. Each color represents a cluster of sequences as determined by k-means clustering of the Euclidean MDS-derived values.

Total RNA was isolated from BM PCs and total PBMCs of three adult NZW rabbits. IgG heavy chain and Igκ/Igλ light chain cDNAs were amplified by 5′ RACE using primers that annealed respectively to the CH1 or CK/Cλ constant region directly 3′ of the J segment (Table S1), and the resulting amplicons were sequenced by Roche 454 sequencing. 172,126 high quality reads corresponding to 88,830 unique heavy chain sequences across the three rabbits were obtained (Table 1). Germline VH usage was determined with IgBLAST [33] alignments using a custom database that included NZW rabbit germline sequences compiled from a number of sources [8], [13], [23], [24], [25], [26], [27], [28], [29] (see Materials and Methods). For the VHa sequences in all three rabbits, >99% were of the a3 allotype, strongly indicating that the cohort of NZW rabbits examined here is homozygous a3/a3 at the IgH locus. However, the IgBLAST alignments revealed a non-normal distribution of VH germline alignment scores (Figure S1A). Based on an analysis by Gertz et al. [7] revealing a number of unannotated germline elements in an a1/a2 Thorbecke rabbit, we hypothesized that the NZW rabbit germline database may be incomplete and thus lack the germline V gene sequences for these poorly scoring Ig alignments. MDS [34], a space-based method that has been used to identify patterns in distance matrices derived from multiple sequence alignments (MSAs) of large biological sequence data sets [31], [35], [36], was employed to deduce putative germline V gene segments. MDS allows MSA distance matrices to be analyzed in Euclidean space, facilitating k-means clustering [37] of the sequences. In the case of somatically mutated Ig V gene sequences, the consensus sequence of each of these k-means defined clusters represents a putative germline V gene sequence. Figure 1 shows the MDS and k-means clustering of the poorly aligned VH gene sequences (higher than 30 nt differences from the nearest VH germline) in the NZW rabbit repertoire. For each of the three rabbits, four distinct VH clusters were identified. Each cluster of VH sequences was extracted and aligned, and the consensus sequence for each of the four clusters was compared across the three rabbits. Each of the four VH consensus sequences (Table S2) matched identically across all three rabbits, strongly supporting our hypothesis that the poorly aligned sequences are derived from unannotated germline VH elements encoded in the NZW rabbit genome.

**Table 1:** Summary of 454 NGS data.

Sample	reads	unique VH/VL amino acid sequences	unique CDRH3/CDRL3
Rabbit rab1 PBMC VH	16102	9447	5525
Rabbit rab1 Bone marrow PC VH	31136	19044	5954
Rabbit rab2 PBMC VH	24251	13459	7220
Rabbit rab2 Bone marrow PC VH	76510	34762	11564
Rabbit rab3 Bone marrow PC VH	24127	12118	5958
Rabbit rab1 Bone marrow PC VL	24489	10446	5629
Rabbit rab2 Bone marrow PC VL	17155	7487	4465
Rabbit rab3 Bone marrow PC VL	23761	12581	7139

**Figure 2:**
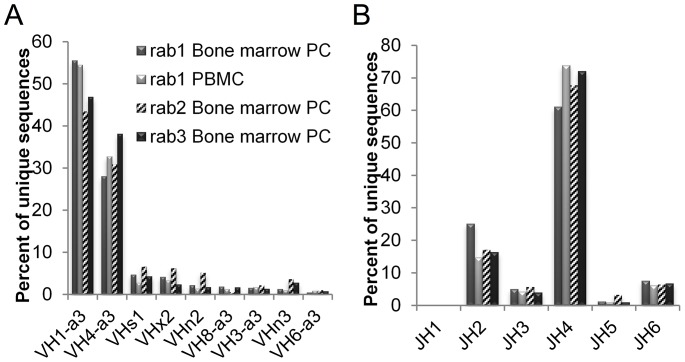
Heavy chain germline gene usage. (A) VH and (B) JH germline usage of unique IgG sequences in rabbits. Sample sizes of unique sequences: rab1 Bone marrow PC, N = 19,291; rab1 PBMC, N = 9,235; rab2 Bone marrow PC, N = 10,148; rab3 Bone marrow PC, N = 12,107.

The four putative germline sequences identified by MDS and k-means clustering were searched by BLASTn to identify homology to publicly available rabbit genomic and transcript sequences (Table 2). For three of the four putative VH germline sequences, NZW rabbit genomic or transcript sequence matches were found that were identical or within 1–3 nucleotide differences. The closely matching transcript sequences (AY676808, AF264452, and AF264440) were derived from rabbits that have a ligated appendix (LigApx) [14], [38], which effectively eliminates SHM and gene conversion. Three of the four putative germline sequences contained a ^70^WVN^72^ motif, consistent with VHa-negative (VHn) immunoglobulins (VHa sequences have a ^70^WAK^72^ motif), while one sequence (VHs1) had a ^70^SVK^72^ motif, which is predominant in VHs immunoglobulins (which are also VHa-negative) and ancestral to hares [39]. VHx2 was highly identical (281/288 nt) to the VHx32 allele previously annotated [29] and may represent a distinct VHx allele (hence its designated ID). These four new putative germline sequences in the NZW rabbit were added to our existing NZW rabbit germline database (see Materials and Methods for full description) and using this updated database, IgBLAST was used to assign VH and JH germline usage (Figure 2). Consistent with earlier observations [2], [19], the VH1 gene is heavily utilized in all three rabbits, as is the VH4 gene, which is >97% identical to VH1. The VHa-negative sequences (combined) account for 12%, 22%, and 11% of the total IgG sequences in rab1, rab2 and rab3 respectively. All three rabbits also exhibit highly restricted JH usage, with JH4 accounting for 60–70% of the IgG repertoire.

**Table 2:** BLASTn results of the four putative VH germline sequences identified by MDS and k-means clustering.

VH germline (query sequence)	NCBI GenBank accession number	mismatches/total length (nt)	Source of highly identical BLASTn sequence match
VHn3	M12386	3/288	Putative VH from liver genomic DNA
	NW_003160066	4/288	Unplaced genomic scaffold in Thorbecke rabbit
			
VHs1	JN131896	2/285	Genomic VDJ recombinant from immature B cell
	AY676808	1/285	mRNA from peripheral B cell in LigApx rabbit
	NW_003161050	3/285	Unplaced genomic scaffold in Thorbecke rabbit
			
VHx2	AF264452	0/288	mRNA from peripheral B cell in LigApx rabbit
	AF264440	0/288	mRNA from peripheral B cell in LigApx rabbit
	NW_003159519	3/288	Unplaced genomic scaffold in Thorbecke rabbit
			
VHn2	AF245499	10/288	mRNA from rabbit bone marrow and spleen

Each NCBI GenBank accession number is a sequence identified by BLASTn as a highly homologous sequence to the query putative VH germline sequence.

### Vκ and Jκ usage in the rabbit

Similar to mice, rabbits utilize the kappa light chain isotype at a much higher frequency than the lambda isotype [40]. We amplified the light chain repertoire from BM PCs in all three rabbits using 5′ RACE and sequenced the VL region using Cκ and Cλ specific primers. A total of 65,405 high quality reads and 30,514 unique sequences across the three rabbits were obtained (Table 1). As expected, the utilization of lambda light chain sequences sets was very low (<1%). Rabbit immunoglobulin kappa light chains have four allotypes: b4, b5, b6, and b9 [41]. For each of the three rabbits examined here, >98% of the unique VL sequences were of the b4 allotype, indicating this cohort of NZW rabbits was b4/b4 homozygous. Similar to the results of the VH IgBLAST alignments, Vκ gene alignment scores also revealed a non-normal distribution, with a group of sequences exhibiting significantly lower alignment scores as compared to the bulk of the Vκ sequences (Figure S1B). These poorly aligned sequences were examined more closely by MDS and k-means clustering as described above and in the Materials and Methods, and four new Vκ clusters were identified (Figure S2). Two of the four putative Vκ germline sequences, NZWk57r and NZWk155g (Table S2), were utilized in all three rabbits. NZWk57r and NZWk155g have also been detected in non-functional light chain sequences (VJ junction out-of-frame) in the bone marrow of a 1 day old b5/b5 NZW rabbit (i.e. early development when naive, unmutated Ig sequences are common in the rabbit) [11]. For the other two putative Vκ germlines (Table S2), one was identified only in the rab2 and rab3 rabbits (NZWk807y), while the other was identified only in the rab2 rabbit (NZWk529g). Nonetheless, all four cluster consensus sequences were also found by BLASTn analysis as either exact matches or differing by only 1 nt (NZWk807y) from previously identified germline genes in the Thorbecke inbred rabbit.

**Figure 3:**
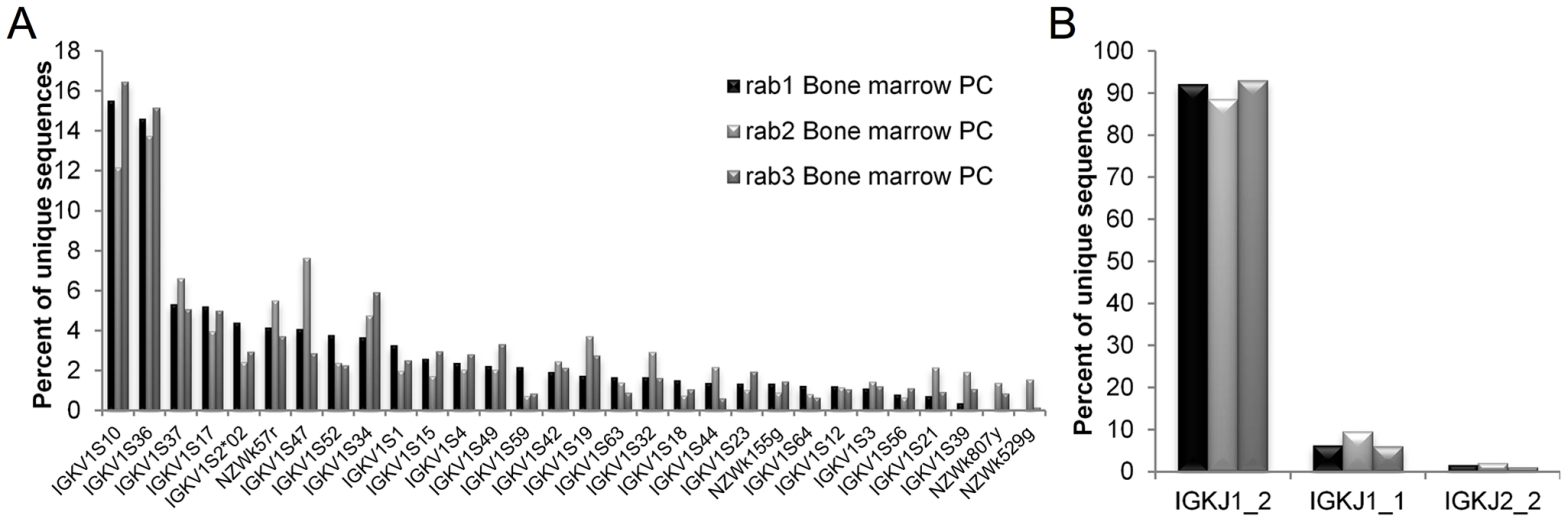
Light chain germline gene usage. (A) Vκ and (B) Jκ germline usage of unique kappa light chain sequences in rabbits. Sample sizes of unique sequences: rab1 Bone marrow PC, N = 10,446; rab2 Bone marrow PC, N = 7,481; rab3 Bone marrow PC, N = 12,580. Germline gene IDs are as listed in the IMGT database.

The four putative Vκ sequences were added to our existing NZW IgBLAST database, which was then used to assign germline Vκ usage (Figure 3). Contrary to the sharp germline restriction seen in the VH gene repertoire, Vκ gene usage is very diverse, with the top germline gene segment used at ∼10–20% and 30 Vκ germlines utilized at least >1% (of total unique Vκ sequences) across the three rabbits. Jκ germline usage, on the other hand, is mostly restricted to the IGKJ1_2 gene (∼90%) and to a very small extent IGKJ1_1 and IGKJ2_2.

### Characterization of the CDRH3 and CDRL3 in the rabbit IgG repertoire as compared to other species

**Figure 4:**
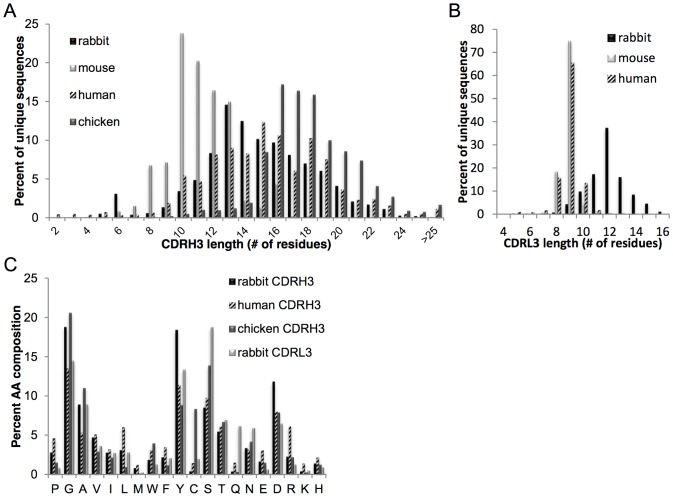
Characterization of the CDRH3 and CDRL3 in rabbit as compared to other species. (A) CDRH3 lengths, (B) CDRL3 lengths, and (C) CDRH3 and CDRL3 amino acid composition. All data shown here is derived from unique heavy chain or light chain sequences. Sample sizes of unique IgG/IgY sequences: mouse heavy chain, N = 2,762; rabbit heavy chain, N = 29,439; rabbit light chain, N = 10,446; human heavy chain, N = 2,948; chicken heavy chain, N = 231,165.

In addition to the rabbit NGS data set, we also analyzed human [42], mouse [18], and chicken NGS data sets to compare and contrast repertoire characteristics across species. For the chicken, we obtained 320,468 high quality VH sequence reads (231,165 unique VH amino acid sequences) from the splenic B cell repertoire of a white leghorn chicken using the Illumina MiSeq 2×250 NGS platform. A comparison of the CDRH3 length distribution is shown in Figure 4. Rabbit IgG CDRH3 lengths are intermediate (mean  = 14.8±3.6 aa, mode  = 13 aa) relative to mice (mean  = 11.1±2.0 aa, mode  = 10 aa), humans (mean  = 15.3±4.0 aa, mode  = 15 aa), and chickens (mean  = 17.9±2.8 aa, mode  = 16 aa). The length distribution of the CDRH3 for all unique IgG sequences was similar across all three rabbits (Figure S3). For CDRL3, mice and humans both exhibit very little junctional diversity and are severely restricted in length, with the vast majority of CDRL3s for both species being 9±1 amino acids (Figure 4); However, the rabbit exhibits significant junctional diversity in the CDRL3, with a wide distribution of CDRL3 lengths (range: 5aa–16aa) and a much greater mean length, equal to 12±1.6 aa.

The amino acid composition of the rabbit Ig CDRH3 is dominated by tyrosine (Y), glycine (G), and aspartate (D) which together represent half (49%) of the amino acid usage in the CDRH3 loop (Figure 4), while the top five amino acids used (GYDAS) represent a full two-thirds (66%) of the amino acid usage. In that regard, the overall amino acid utilization in the rabbit is highly similar to the other species, consistent with earlier observations [43] that the average hydrophobicity of CDRH3—and, hence, the center of the antigen binding site—is conserved across evolution to be slightly hydrophilic and enriched for glycine, serine and tyrosine. Nevertheless, when compared to other species, the CDRH3 amino acid composition in rabbits does show some distinct features. Human CDRH3s use glycine and tyrosine at a much lower frequency than that seen in rabbits. Chicken CDRH3s have less tyrosine (∼2-fold less than rabbits) but utilize much higher cysteine content (∼5–10-fold higher than humans or rabbits). The higher utilization of Cys residues in the chicken CDRH3 repertoire has previously been shown to be important for stabilizing (by disulfide bonds) the longer CDRH3 loops seen in chickens [44]. The amino acid utilization of the rabbit CDRL3 is also shown in Figure 4 for comparative purposes.

### Diversification of the rabbit IgG repertoire by SHM and gene conversion

The rabbit Ig repertoire is known to undergo extensive AID-mediated mutagenesis (via both SHM and gene conversion) early on in development when the antigen-inexperienced naïve B cell repertoire migrates from the bone marrow to the GALT [6]. Earlier studies with rabbits lacking an established gut microflora demonstrated significantly reduced levels of AID-mediated diversification of the repertoire, with most Ig having sequences that approximate the germline elements from which they are derived [14], [38].

**Figure 5:**
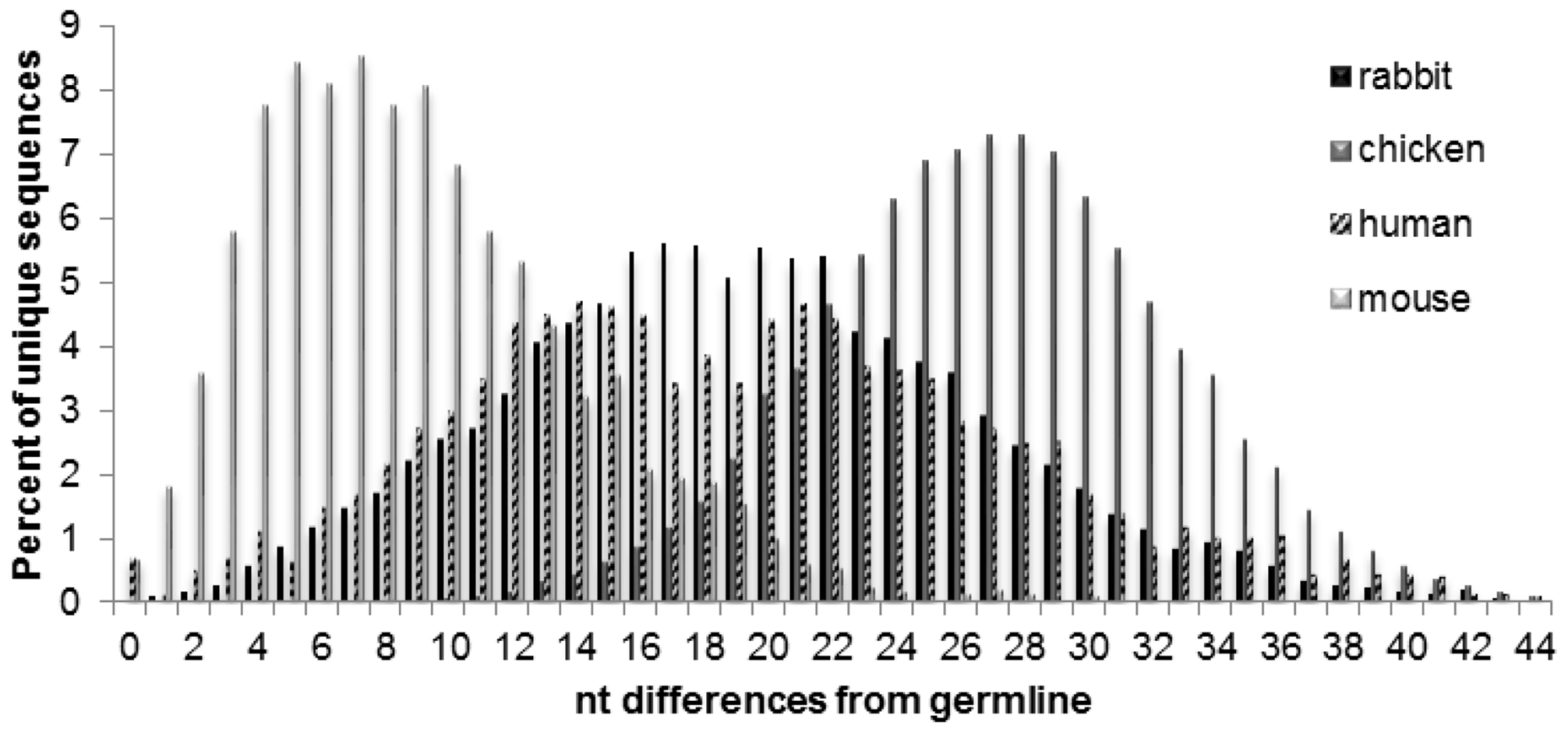
Diversification of the immunoglobulin repertoire – comparison of overall levels of nucleotide changes in the VH sequences across four species. The number of nucleotide differences from the nearest (assigned) VH germline derived from IgBLAST alignments as discussed in the text for rabbits.

We compared the overall level of mutation (combined SHM and gene conversion) within the IgG repertoires of rabbits, chicken, mice and humans (Figure 5). The mutational load varied as follows: chicken>rabbit ≈ human>mouse. It should be noted that the reported mutational load is a combination of both biological processes mediated by AID and inherent PCR/sequencing error, which has been reported to be approximately 1% for both 454 GS-FLX [45] and Illumina MiSeq sequencing [46]. To determine the relative contribution of gene conversion to the diversification of the primary repertoire, we developed a script that searches Ig sequences for tracts of putative gene conversion events. Gene conversion tracts are detected as a contiguous block of nucleotides within a query Ig sequence that closely matches a different germline element (e.g. not the query's assigned germline element) in the IgBLAST database. Additionally, to rule out possible PCR template switching artifacts, the gene conversion tracts were required to be bound on each end by positions (tracts) that match the query's assigned VH germline sequence (i.e. the gene conversion event was not contiguous with the 5′ or 3′ ends of the sequence). Additionally, minimum scoring and p-value thresholds were applied as described in the methods. Strict statistical thresholds were set to ensure that the identified gene conversion events were highly significant and not attributed to high loads of point mutation. For these reasons, the reported frequencies of gene conversion events should be considered as a lower bound of the actual biological frequencies (Table 3).

**Table 3:** Gene conversion analysis: comparison of the frequencies, average scores, and average GC tract lengths in four different species.

Organism/sample	unique seqs	% with GC	max GC score	avg tract (nt)
rabbit BM PC IgG	10680	23	17	59±36
rabbit BM PC Igκ	2062	32	19	86±48
chicken Spleen IgY	10000	70	23	79±57
mouse BM PC IgG	946	0.1	5	6
human PBMC IgG	1028	2.5	7	39±27

The vast majority of unique chicken IgY sequences examined (70%) display evidence of gene conversion events. In rabbits, 23% of IgG sequences and 32% of Igκ sequences were the products of gene conversion. There have been previous, although somewhat controversial, indications suggesting gene conversion occurs in humans and mice as well, albeit at a much lower frequency [47], [48], [49]. We find that, in the mouse, putative gene conversion events are nearly absent, with an estimated frequency of 0.1% of all unique IgG sequences. Whereas an earlier analysis of gene conversion in a small set of human IgG sequences indicated that ∼7% (8 out of 121) display evidence of having undergone gene conversion [49], our present analysis of a much larger data set revealed a lower frequency of 2.5%. We note that, in humans and mice, the low p-values (p<0.05) in the detection of gene conversion events suggest that these are high confidence identifications despite the fact that the average tract lengths detected were significantly lower than those in the rabbit and chicken (Table 3).

**Figure 6:**
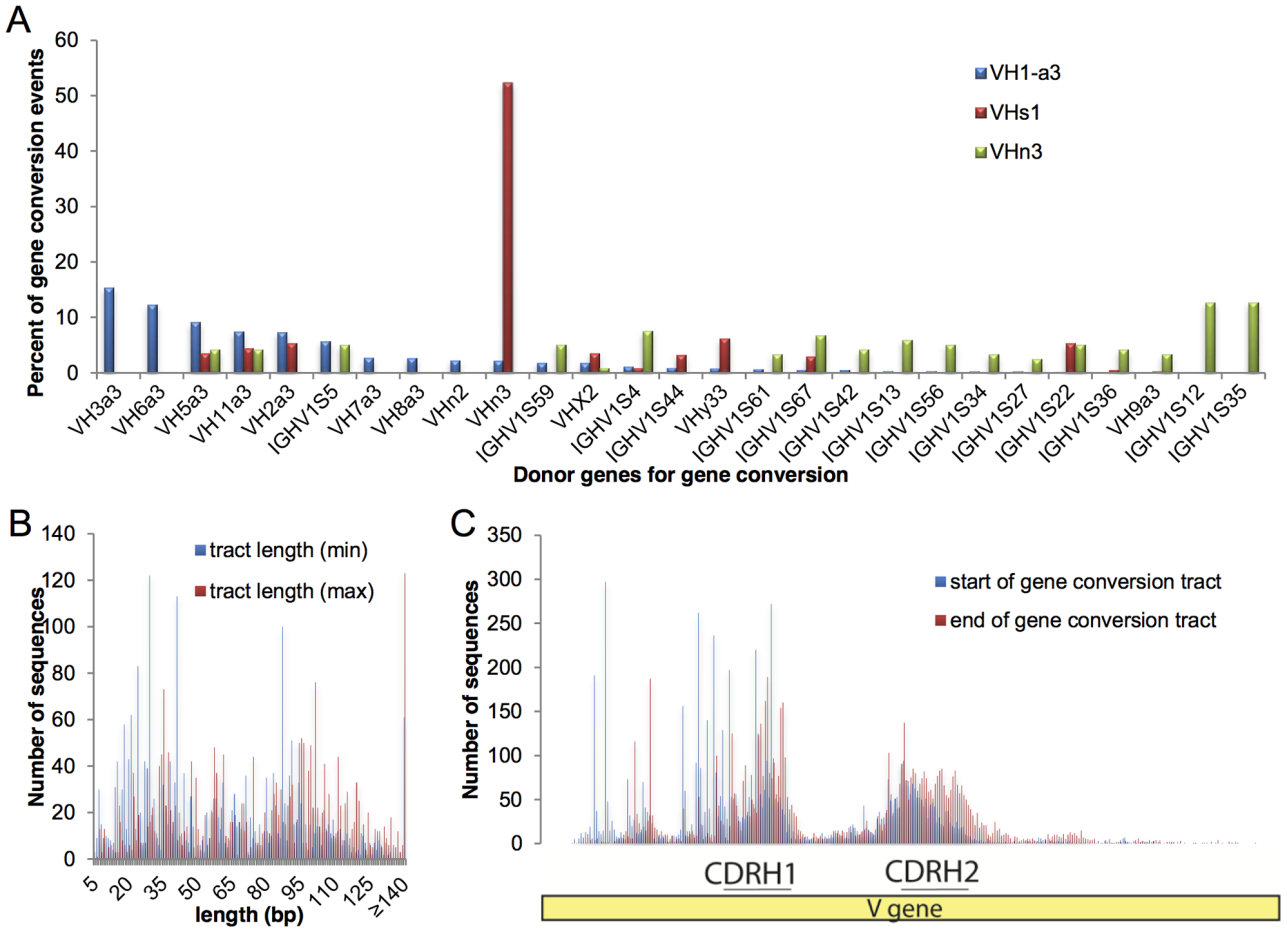
Gene conversion analysis. (A) Examination of gene conversion donor VH germline usage for recipient query sequences. For the recipient query sequences examined here, the germline VH usage (i.e. the original recombined V gene) was either VH1-a3, VHs1, or VHn3. (B) Gene conversion tract lengths (i.e. lengths of recombined fragments). See Materials and Methods for description of short (min) versus long (max) tract lengths. (C) The nucleotide positions along the VH gene sequence where the gene conversion recombination events start and stop. Start and end positions are based upon the short (min) tract as described in the Materials and Methods.

The frequencies of donor germline VH usage for gene conversion in the rabbit are largely unknown. Figure 6 shows the donor germline VH usage for query sequences that were assigned by IgBLAST to one of three heavily utilized germline VH gene segments in the rabbit (VH1, VHs1, and VHn3). Because gene conversion occurs through homologous recombination, the frequency is heavily dependent on donor VH sequence homology and proximity. High homology donor VH genes directly upstream of the assigned VH reference (e.g. the VH germline originally used during VDJ recombination) are expected to be used in gene conversion more frequently than donor genes that are more distal or less homologous. The donor germline usage for VH1 is consistent with this expectation, with the genes directly upstream being used as donors for gene conversion more frequently than those more distal to VH1. The two VHa-negative sequences (VHs1 and VHn3) have very different patterns of germline VH donor usage. The genomic location and organization of these two VHa-negative elements are not known, but it is clear that VHs1 must be downstream of VHn3 as it heavily utilizes VHn3 as a donor sequence for gene conversion.

The tract lengths and start/end residue numbers of the gene conversion events for assigned VH1 sequences are shown in Figure 6B and 6C. The majority of gene conversion tracts in rabbit IgG are under 30 bp in length, although some identified tracts are much longer (>120 bp). As expected for AID-mediated events, the gene conversion tracts have start and end positions that mostly localize to the CDRH1 and CDRH2 regions of the V genes, where a number of conserved AID hotspot motifs are located. These CDRs, along with CDRH3, constitute a large amount of the paratope in antibodies and thus are strongly mutated and selected during the affinity maturation process.

## Discussion

The vertebrate adaptive immune system is unparalleled in its ability to sample the depths of protein sequence space for the production of high-affinity antibodies endowed with exquisite specificity. Not only are antibodies extremely useful in the lab as affinity reagents, but they also represent the fastest growing sector of the biologics drug market, with annual global sales for monoclonal antibodies approaching $50 billion [50]. This has resulted in an increased interest for mining the antibody repertoires within vertebrates in a systematic, high resolution manner, something afforded by increasingly economical NGS technologies that enable the collection of thousands to millions of DNA sequences in a single sequencing run. Several species' Ig repertoires have been characterized by NGS to date [9], [18], [51], [52], [53], [54]. In this report, we used 5′ RACE-amplification of rabbit IgG and Igκ/Igλ transcripts, followed by NGS and bioinformatics analyses, to elucidate key features of the repertoire. We provide evidence that the existing rabbit germline VH gene database, as annotated from a number of sources [8], [13], [23], [24], [25], [26], [27], [28], [29] (see Materials and Methods), is incomplete. This was not surprising based on previous estimations of the number of Ig germline elements in the rabbit and also a very recent survey of Ig germline elements detected in the genome of a Thorbecke inbred rabbit [7].

There are typically two types of approaches for examining sequence relationships in the multiple alignments of homologous sequences: (1) tree-based methods (e.g. phylogenetics) and (2) space-based methods that, unlike phylogenetics, do not infer a hierarchical or a specific structure within the sequence alignment. For the assignment of germline sequences, space-based methods provide a statistical framework for comparing and clustering the sequences based on pairwise identities or similarities. MDS is a space-based method that allows the pairwise distances in the multiple sequence alignment to be reduced to a small number of principle components that aid in clustering the data within Euclidean space. This type of analysis applied to large Ig sequence data sets allows accurate genotyping of the germline elements within the species simply based upon the detection of highly frequent shared polymorphisms observed across individuals [55]. We show that MDS combined with k-means clustering provides an efficient approach towards discovery of new Ig germline elements in NGS data sets, even with repertoires that exhibit high loads of mutations, as is the case with the rabbit IgG repertoire where a large fraction of Ig sequences deviate significantly from the germline due to gene conversion events. MDS combined with k-means clustering could be successfully applied to a multitude of species for which the germline Ig loci are poorly annotated.

The large sample size provided by NGS also allows the diversification mechanism of Ig repertoires to be analyzed in great detail. We show that in the rabbit, the frequency of gene conversion is significantly lower than in the chicken. Consistent with this finding, it had been previously reported that chickens depend on gene conversion as the primary mechanism of Ig diversification and that SHM play a smaller role [56]. In rabbits, the chromosomal organization of VH gene elements is quite complex, with many VH germline genes located in genomic regions far removed from the commonly utilized VH1 germline gene. This may effectually limit the relative frequency of gene conversion, as gene conversion of VH1 is limited mostly to those donor genes directly upstream. Further, several of these upstream donor genes are functional, whereas in chickens there exists a single functional germline VH and a pool of upstream pseudogenes that are used exclusively as donor genes for gene conversion. Interestingly, and consistent with earlier data [47], we report a detectable amount of gene conversion in the human IgG repertoire, but not in the mouse. The gene conversion tract lengths are significantly lower in the expressed human IgG repertoire as compared to the rabbit and chicken, but nonetheless are of high statistical confidence (p<0.05). This finding argues that gene conversion needs to be explicitly taken into account in the analysis of the antibody repertoire.

## Supporting Information

Figure S1 **IgBLAST database alignment performance.** Comparison of IgBLAST alignment performance before and after addition of putative (A) VH and (B) Vκ germline sequences identified by MDS and k-means clustering. Before addition of the newly annotated germline sequences (IgBLAST database v.1), a large shoulder of very high ‘mutation’ load is evident in the IgBLAST alignments. After addition of the germline sequences identified by MDS and k-means clustering (IgBLAST database v.2), the vast majority of the sequences with high ‘mutation’ load now align to one of the new germline annotations and thus have a lower amount of nt differences from the nearest VH germline sequence.
(TIF)

Figure S2 **MDS and k-means clustering of low scoring Vκ-aligned sequences in rab2 rabbit bone marrow PC IgG.** The first three components of the MDS are shown here, comparing (A) PC1 v. PC2 and (B) PC1 v. PC3. Included in yellow are all the germline Vκ sequences in the original IgBLAST database (v.1). The population in blue represents light chain sequences that cluster with germline Vκ already existing in the original IgBLAST database (v.1). rab1 and rab3 MDS and k-means clustering produced similar results, but unlike with VH clusters, not all four identified clusters were observed across all three rabbits (as detailed in the main text). Here, for example, the rab2 rabbit only has three new k-means clusters (in red, green, and gray). Due to the high identity (94%) between NZWk155g and NZWk57r (both part of the red cluster here), k-means was unable to separate these into two distinct clusters for the rab2 analysis.
(TIF)

Table S1 **Primers used to amplify IgH and Igκ/Igλ repertoires.**
(DOCX)

Table S2 **NZW rabbit VH and Vκ germline sequences identified by MDS and k-means clustering.**
(DOCX)
