# Supplementary figures and images for: Systematic Characterization and Comparative Analysis of the Rabbit Immunoglobulin Repertoire
Source: PLoS One. 2014 Jun 30;9(6):e101322. doi: 10.1371/journal.pone.0101322 (PMC4076286; doi:10.1371/journal.pone.0101322)

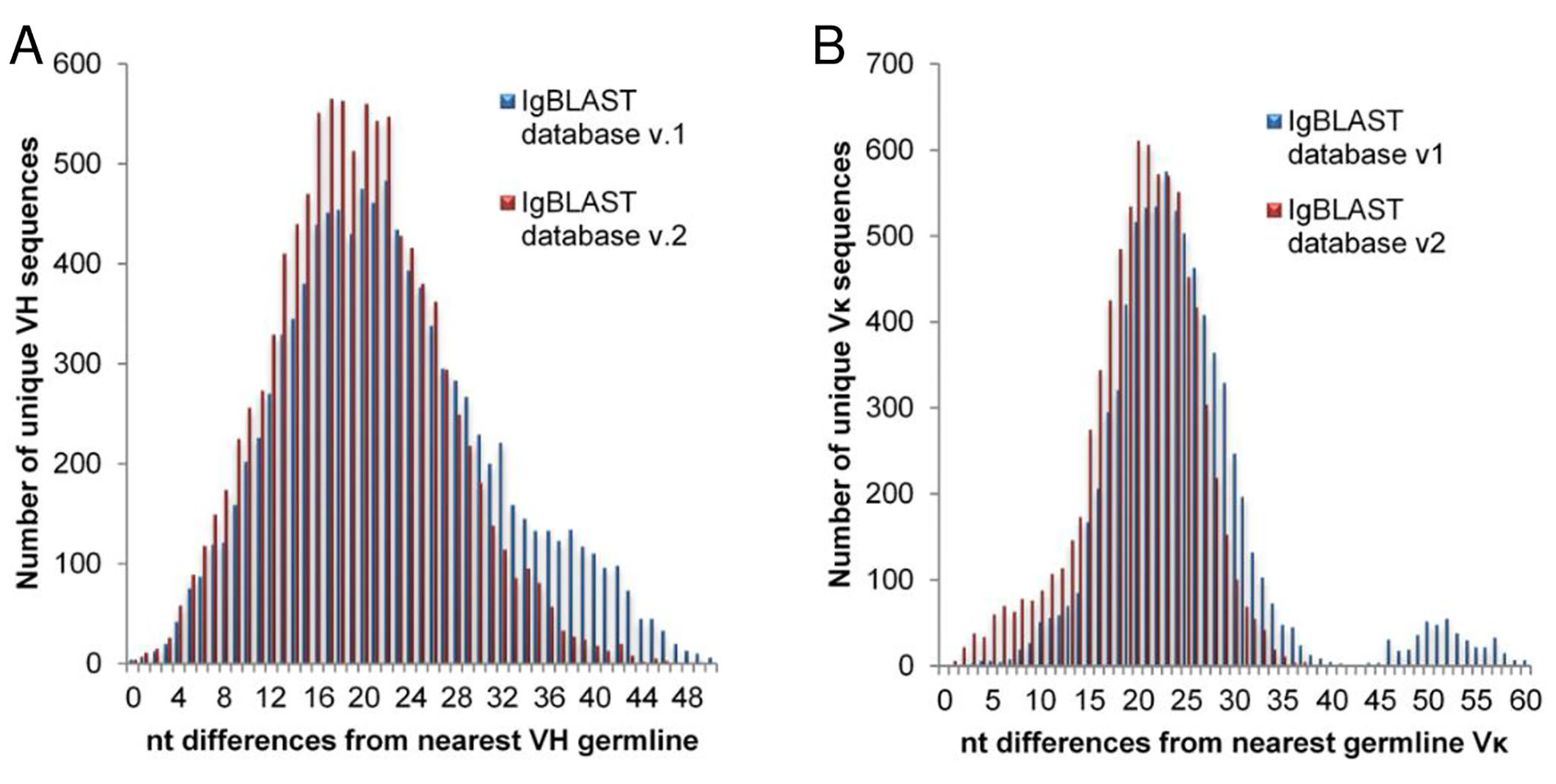

Supplement: Figure S1 — IgBLAST database alignment performance. Comparison of IgBLAST alignment performance before and after addition of putative (A) VH and (B) Vκ germline sequences identified by MDS and k-means clustering. Before addition of the newly annotated germline sequences (IgBLAST database v.1), a large shoulder of very high ‘mutation’ load is evident in the IgBLAST alignments. After addition of the germline sequences identified by MDS and k-means clustering (IgBLAST database v.2), the vast majority of the sequences with high ‘mutation’ load now align to one of the new germline annotations and thus have a lower amount of nt differences from the nearest VH germline sequence. (TIF) [file pone.0101322.s001.tif]

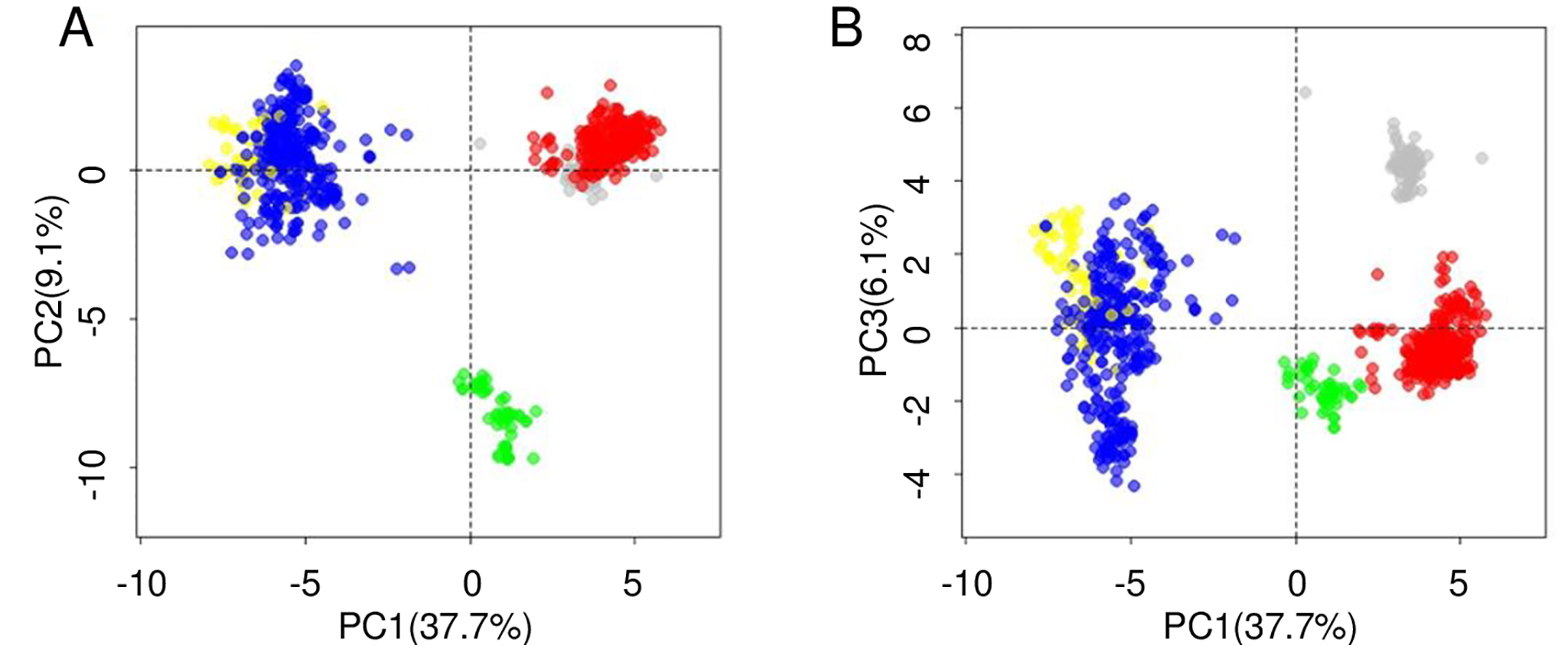

Supplement: Figure S2 — MDS and k-means clustering of low scoring Vκ-aligned sequences in rab2 rabbit bone marrow PC IgG. The first three components of the MDS are shown here, comparing (A) PC1 v. PC2 and (B) PC1 v. PC3. Included in yellow are all the germline Vκ sequences in the original IgBLAST database (v.1). The population in blue represents light chain sequences that cluster with germline Vκ already existing in the original IgBLAST database (v.1). rab1 and rab3 MDS and k-means clustering produced similar results, but unlike with VH clusters, not all four identified clusters were observed across all three rabbits (as detailed in the main text). Here, for example, the rab2 rabbit only has three new k-means clusters (in red, green, and gray). Due to the high identity (94%) between NZWk155g and NZWk57r (both part of the red cluster here), k-means was unable to separate these into two distinct clusters for the rab2 analysis. (TIF) [file pone.0101322.s002.tif]
