# Supplementary material for: Systematic Characterization and Comparative Analysis of the Rabbit Immunoglobulin Repertoire
Source: PLoS One. 2014 Jun 30;9(6):e101322. doi: 10.1371/journal.pone.0101322 (PMC4076286; doi:10.1371/journal.pone.0101322)
Supplement: Table S1 — Primers used to amplify IgH and Igκ/Igλ repertoires. (DOCX) [file pone.0101322.s003.docx]

| **Table S1**. Primers used to amplify IgH and Igκ/Igλ repertoires | |  |
| --- | --- | --- |
|  |  |  |
| **Primer Name** | **Sequence** | **Description of use** |
| RIGHC1 | CAGTGGGAAGACTGACGGAGCCTTAG | Rabbit IgG CH1 reverse V_H_ primer mix (equimolar) |
| RIGHC2 | CAGTGGGAAGACTGATGGAGCCTTAG | Rabbit IgG CH1 reverse V_H_ primer mix (equimolar) |
| RIGκC1 | TGGTGGGAAGAKGAGGACAGTAGG | Rabbit Igκ reverse primer mix (90% of mix) |
| RIGκC2 | TGGTGGGAAGAKGAGGACACTAGG | Rabbit Igκ reverse primer mix (5% of mix) |
| RIGκC3 | TGGTGGGAAGAKGAGGACAGAAGG | Rabbit Igκ reverse primer mix (5% of mix) |
| RIGλC1 | CAAGGGGGCGACCACAGGCTGAC | Rabbit Igλ reverse primer mix (equimolar) |
| RIGλC2 | GTGAAGGAGTGACTACGGGTTGACC | Rabbit Igλ reverse primer mix (equimolar) |
| RIGλC3 | GAGGGGGTCACCGCGGGCTGAC | Rabbit Igλ reverse primer mix (equimolar) |
| Chicken VH1 | GCCGTGACGTTGGACGAGTCC | Chicken VH1 forward primer |
| Chicken IgY | GGAGGAGACGATGACTTCGGTCCC | Chicken IgY reverse primer |
