# Supplementary material for: Systematic Characterization and Comparative Analysis of the Rabbit Immunoglobulin Repertoire
Source: PLoS One. 2014 Jun 30;9(6):e101322. doi: 10.1371/journal.pone.0101322 (PMC4076286; doi:10.1371/journal.pone.0101322)
Supplement: Table S2 — NZW rabbit VH and Vκ germline sequences identified by MDS and k-means clustering. (DOCX) [file pone.0101322.s004.docx]

| **Table S2**. NZW rabbit VH and Vκ germline sequences identified by MDS and k-means clustering | |
| --- | --- |
|  |  |
| **germline ID** | **Germline nucleotide sequence** |
| VHs1 | CAGGAGCTGGTGGAGTCTGGAGGGGGTCTGGTCCAGCCGGGGGAATCCCTGAAACTCTCCTGCAAAGCCTCTGGAATCGACTTCAGTAGCTATGGCATTAGCTGGGTCCGCCAGGCTCCAGGGAAGGGGCTGGAGTGGATCGCATACATTTATCCTGGTTTTGGTATCACAAACTACGCGAACTCTGTGAAGGGCCGATTCACCATCTCCAGCGACAACGCCCAGAACACGGTGTTTCTGCAAATGACCAGTCTGACAGCCTCGGACACGGCCACCTATTTCTGT |
|  |  |
| VHx2 | CAGGAGCAGCTGGTGGAGTCCGGGGGAGGCCTGGTCCAGCCTGGGGGATCCCTGAAACTCTCCTGCAAAGCCTCTGGATTCGACTTCAGTAGCTATGGAGTGAGCTGGGTCCGCCAGGCTCCAGGGAAGGGGCTGGAGTGGATCGGGTACATTGATCCTGTTTTTGGTAGCACATACTACGCGAGCTGGGTGAATGGCCGATTCACCATCTCCAGCCACAACGCCCAGAACACGCTGTATCTGCAACTGAACAGTCTGACAGCCGCGGACACGGCCACCTATTTCTGT |
|  |  |
| VHn3 | CAGGAGCAGCTGAAGGAGACCGGGGGAGGCCTGGTCCAGCCTGGGGGATCCCTGACACTCTCCTGCAAAGCCTCTGGATTCGACTTCAGTAGCTACTACATGAGCTGGGTCCGCCAGGCTCCAGGGAAGGGGCTGGAGTGGATCGGAATCATTTATGCTGGTAAAGGTAGCACAGACTACGCGAGCTGGGTGAATGGCCGATTCACCATCTCCAGCGACAACGCCCAGAACACTGTGGATCTTCAAATGAACAGTCTGACAGCGGCGGACACGGCCACCTATTTCTGT |
|  |  |
| VHn2 | CAGGAGCAGCTGGTGGAGTCTGGAGGGGGTCTGGTCACGCTTGGGGGATCCCTGAAACTCTCCTGCAAAGCCTCTGGAATCGACTTCAGTAGCTATGGCATTAGCTGGGTCCGCCAGGCTCCAGGGAAGGGGCTGGAGTGGATCGCATACATTTATCCTGATTATGGTAGCACAGACTACGCGAGCTGGGTGAATGGCCGATTCACCATCTCCCTCGACAACGCCCAGAACACGGTGTTTCTGCAAATGACCAGTCTGACAGCCGCGGACACGGCCACCTATTTCTGT |
|  |  |
| NZWk57r | GCCATTGAGATGACCCAGTCTCCACCCTCCCTGTCTGCATCTGTGGGAGAAACTGTCAGGATTAGGTGCCTGGCCAGTGAGGACATTTACAGTGGTATATCCTGGTATCAACAGAAGCCAGGGAAACCTCCTACACTCCTGATCTATGGTGCATCCAATTTAGAATCTGGGGTCCCACCACGGTTCAGTGGCAGTGGATCTGGGACAGATTACACCCTCACCATTGGCGGCGTGCAGGCTGAAGATGCTGCCACCTACTACTGTCTAGGCGGTTATAGTTATAGT |
|  |  |
| NZWk155g | GCCATTGAGATGACCCAGTCTCCACCCTCCCTATCTGCATCTGTGGGAGAAACTGTCAGGATTAGGTGCCTGGCCAGTGAGTTCCTTTTTAATGCTGTATCCTGGTACCAACAGAAGCCAGAGAAACCTCCTACACTCCTGATCTCTGGTGCATCCAATTTAGAATCTGGGGTCCCACCACGGTTCAGTGGCAGTGGATCCGGGACAGATTACACCCTCACCATCGGCGGCGTGCAGGCTGAAGATGTTGCCACCTACTACTGTCTAGGCGGTTATAGTGGTAGT |
|  |  |
| NZWk529g | GCCATCCAGATGACCCAGTCTCCATCCTCCCTGGCTGCATCTGTGGGAGACACAGTCACCATCACTTGTAAGGCCAGTGAGGACATTGGTTATGGGTTAAACTGGTATCAGCAGAAACTAGGGATAGCTCCTAAGCTCCTGATCTATGGGGCAAACACTTTAGAATCTGGGGTCCCATCGAGGTTCAGTGGCAGCGGATCAGAGACCGATTACACCCTCACCATCAGCAGCGTGCAGGCTGAAGATGCAGGAATTTATTACTGTCAGCAAGGATATAGTACCCCT |
|  |  |
| NZWk807y | GCCATCGAGATGACCCAGTCTCCACCCTCCCTGTCTGCATCTGTGGGAGGCACAGTCACCATAAACTGTCTGGCGAGTGAGAACGTTTACAGTGCTGTAGCCTGGTATCAACAGAAGCCAGGGAAACCTCCTACACTCCTGATCTCTGGTGCATCCAATTTAGAATCTGGGGTCCCACCACGGTTCAGTGGCAGTGGATCTGGGACAGATTACACCCTCACCATCGGCGGCGTGCAGGCTGAAGATGCTGCCACTTACTTCTGTCAAGGGTATAGCAGTTACCCT |
